# A small basic protein from the *brz-brb *operon is involved in regulation of *bop *transcription in *Halobacterium salinarum*

**DOI:** 10.1186/1471-2199-12-42

**Published:** 2011-09-19

**Authors:** Valery Tarasov, Rita Schwaiger, Katarina Furtwängler, Mike Dyall-Smith, Dieter Oesterhelt

**Affiliations:** 1 Max-Planck Institute of Biochemistry, Department of Membrane Biochemistry, Am Klopferspitz 18, 82152 Martinsried, Germany; 2 Institut für Klinische Chemie, Klinikum Schwabing, Am Kölner Platz 1, 80804 Munich, Germany; 3 Max-Planck Institute of Molecular Plant Physiology, Department 2, Am Mühlenberg 1, 14476 Potsdam-Golm, Germany; 4 School of Biomedical Sciences, Charles Sturt University, 2678 NSW, Australia

## Abstract

**Background:**

The halophilic archaeon *Halobacterium salinarum *expresses bacteriorhodopsin, a retinal-protein that allows photosynthetic growth. Transcription of the *bop *(*b*acterio*op*sin) gene is controlled by two transcription factors, Bat and Brz that induce *bop *when cells are grown anaerobically and under light.

**Results:**

A new gene was identified that is transcribed together with the *brz *gene that encodes a small basic protein designated as Brb (bacteriorhodopsin-regulating basic protein). The translation activity of the start codon of the *brb *gene was confirmed by BgaH reporter assays. *In vivo *site-directed mutagenesis of the *brb *gene showed that the Brb protein cooperates with Brz in the regulation of *bop *expression. Using a GFP reporter assay, it was demonstrated that Brb cooperates with both Brz and Bat proteins to activate *bop *transcription under phototrophic growth conditions.

**Conclusions:**

The activation of the *bop *promoter was shown to be dependent not only on two major factors, Bat and Brz, but is also tuned by the small basic protein, Brb.

## Background

*Halobacterium salinarum *is a halophilic archaeon utilizing light to produce ATP via a retinal-based photosynthetic system. The key component of this system is a light-driven proton pump consisting of the integral membrane apoprotein bacterioopsin (OE3106F, VNG1467G) with a covalently attached retinal. Under conditions of low oxygen and high light, bacteriorhodopsin (BR) is highly expressed and forms two-dimensional crystals in the cell membrane, the so-called purple membrane [1,2]. Bacterioopsin is encoded by the *bop *gene, clustered in a locus together with other genes related to its synthesis (additional file 1). One of these is *brp *(*b*acterioopsin-*r*elated *p*rotein) (OE3102R, VNG1465G) [3-5]. Brp, as well as the paralog Blh (*blh *is located 500 kbp from the *bop *locus), are enzymes that convert beta-carotene to retinal [6]. Another gene near *bop *is *crtB1*, which encodes phytoene synthase (CrtB1), an essential enzyme for bacteriorhodopsin production. CrtB1 catalyzes the synthesis of phytoene from the geranylgeranyl pyrophosphate, a key step in the retinal biosynthetic pathway [7]. Also clustered with *bop *are several genes of unknown function, which are conserved among photosynthetic haloarchaea [8]. One of them is *blp *(*b*op-*l*inked *p*rotein, OE3100F, VNG1463G), encoding a protein co-regulated with the *bop *gene [9]. The other three are: *bac *(*b*acterioopsin *a*ssociated *c*haperone, OE3098R, VNG1462G), encoding a paralog of Cdc48, an AAA-ATPase cell division cycle protein; *bap *(*b*acterioopsin *a*ssociated *p*rotein, OE3107F, VNG1468H), a small ORF, following the *bop *gene [8]; and the *OE3095R *gene (*VNG1459H*), coding for a protein of unknown function but possessing a zinc finger like motif that could function in DNA binding [10].

The *brp *gene is transcribed together with *bat*, a gene encoding a transcription factor (*b*acterioopsin *a*ctivator of *t*ranscription, OE3101R, VNG1464G) that activates the *bop *operon [11,12]. The Bat protein contains GAF and PAS/PAC domains, as well as a C-terminal DNA-binding helix-turn-helix motif [7] and induces the *bop *operon in the presence of light and at low oxygen tension [11,13]. Mutations in Bat were suggested to be responsible for the phenotype of constitutive BR-overproduction in the S9 mutant strain of *H. salinarum *[14,7]. Another protein factor regulating *bop *transcription is Brz (bacterioopsin regulating zinc-finger protein, OE3104F, VNG1466H), a small zinc finger protein (60 aa) The *brz *gene is located between the *brp*-*bat *and *bop *operons, and previous work has shown that Brz is not only involved in the regulation of *bop*, but also of *crtB1*, *OE3107F *and *OE3095R *[10]. Since *crtB1 *codes for a phytoene synthase, Brz appears to be a regulator of both the Bop apoprotein and the retinal chromophore production. Such co-regulation is possible by binding to an upstream activator sequence in the promoters of both *bop *and *crtB1 *operons [15,7]. DNA microarray studies of *brz *mutants showed that the regulatory effects of Brz on the *bop *operon are not mediated via Bat. This indicates two possibilities for *bop *regulation: either a regulation of Brz via Bat or the cooperation between Bat and Brz at the protein level [10]. OE3095R, a protein containing a CPxCG-related zinc finger motif could also be involved in a hierarchical *bop *gene regulatory network [10]. Long before the *brz *gene was identified, differences in the nucleotide sequence of this region were reported between mutant S9 and the wild type R1 strain. Sequence differences were also reported downstream of *brz *but before *bop*. Until now, it was not clear if these changes were relevant to the BR overproducing phenotype of strain S9 [3,16]. Besides Bat, *Hbt. salinarum *also carries two homologues of Bat, annotated as *boa2 *and *boa4*, but their functions have not yet been experimentally determined. Adjacent to *boa2 *(*OE3134F*, *VNG1488G*) and *boa4 *(*OE2448F*, *VNG0996G*) are genes encoding small proteins that also contain CPxCG-related zinc finger motifs: OE3131F (VNG1486H, 53 aa) and OE2447F (VNG0995H, 62 aa).

In this study we investigated the functions of two transcription factors, Brz and Bat, in the context of *bop *gene regulation. We show that the *brz *mRNA is bi-cistronic and the additional ORF encodes a small basic protein, Brb. The protein could not be detected by mass spectrometry but the translation activity of start codon of the *brb *gene was confirmed by BgaH reporter assays. The requirement of this protein for *bop *transcription, and the relevance of S9 mutations of *brz *and *brb *to the S9 phenotype, was tested by site-directed mutagenesis *in vivo*. In addition, a cooperation of three proteins, Bat, Brz and Brb, for light dependent activation of the *bop *promoter was demonstrated using a GFP reporter assay.

## Results

### Cooperation of three proteins: Bat, Brz and a small basic protein as partners in the regulation of *bop *transcription

**Figure 1:**
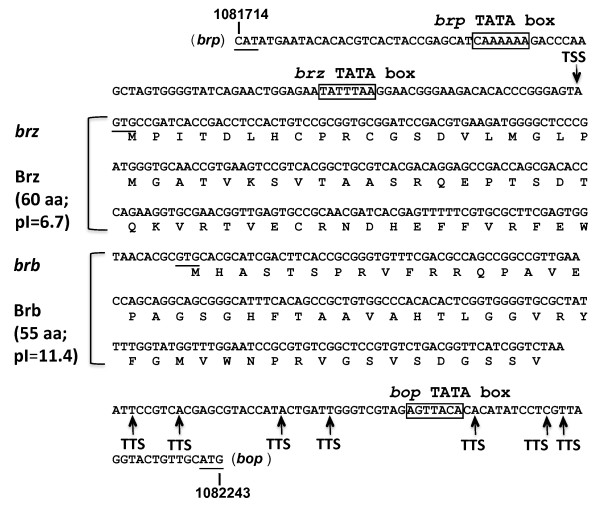
**Operon organization of the *brz *and *brb *genes of *Hbt. salinarum *R1**. The protein sequences are given below the nucleotide sequences. The arrow above the nucleotide line indicates the transcription start site (TSS) and arrows below show transcription termination sites determined by 5'-3'RACE (TTS). The TATA motifs of the *brz*, *brp and bop *genes are indicated by boxes.

In a previous study, Brz was identified as a second regulator of *bop *transcription in *Hbt*. *salinarum *[10]. This changed a simple model of *bop *transcription control from one using only one activator, Bat, to a more complex system with two indispensible activators that probably interact with each other. The same study also showed that the regulatory effects of Brz on *bop *are not mediated via Bat [10]. In the current study, the activation effect of Bat on *bop *via direct regulation of Brz by Bat was excluded by the phenotype of Δ*bat *mutants, in which transcription of *brz *was found not to be down-regulated (data not shown). These results strongly suggest that Bat and Brz cooperate at the protein level. In addition, a new candidate for cooperation with Brz and Bat was found to be encoded by a small ORF on the *brz *mRNA. The existence of this ORF was recognized after 3'-end determination of the *brz *transcript. For this, the mRNA was circularized and the joined ends reverse-transcribed and PCR amplified according to the method described by Brenneis; 2007 [17]. The 3'-ends of the *brz *mRNA were located 173-228 bp downstream of the *brz *stop codon (Figure 1), in some cases downstream of the TATA box of the adjacent *bop *gene. Although mRNA degradation cannot be excluded as a cause of the multiple 3' ends detected by RACE, previous studies have shown that ragged transcription termination is common in Archaea [17]. These results correlate well with the position of the 3'-end of the *brz *mRNA reported previously by Koide; 2009 [18], who used a different approach that averaged the signals from the mRNA population. They located the 3'-end of the message at 226.95 bp from *brz *stop codon. In both cases, there are sufficient nucleotides downstream of the *brz *stop codon to encode a small protein of 5.7 kDa (55 amino acids long) with an alkaline pI of 11.4 (Figure 1). The gene was named *brb *(*b*acteriorhodopsin-*r*egulating *b*asic protein) as, later in the current study, it was found to be involved in regulation of *bop *transcription. According to RT qPCR results, *brz-brb *transcript level is as low as the level of *bat *transcripts (data not shown).

### The *brb *translation start codon is active

**Figure 2:**
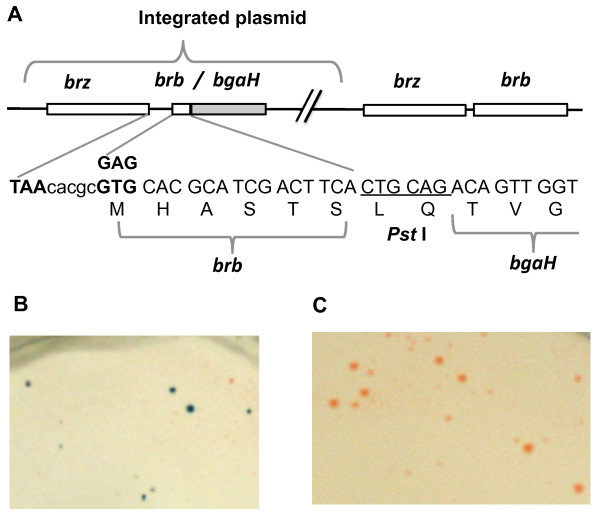
**Test of *brb *translation start codon activity**. Panel A represents the organization of the *brb *and *bgaH *genes in the chromosome of *Hbt. salinarum *after integration of suicide plasmids pVYT1 and pVYT2 (additional file 9). Panel B shows colonies of a strain expressing an active Brb-BgaH fusion protein (insertion of pVYT1) on plates containing X-gal (40 μg ml^-1^). Panel C presents a control mutant lacking β-galactosidase activity as a result of elimination of the *brb *start codon in the *brb-bgaH *gene fusion (insertion of pVYT2).

To test the translation activity of the start codon of the *brb *gene, a mutant producing an N-terminal BgaH fusion protein was constructed by insertion of a suicide plasmid into the *brz-brb *locus. The construct (pVYT1 plasmid) contained the 5'-end of the *brb *gene fused to the *bgaH *reporter gene (lacking its own translational start codon), thus translation could only start from the start codon of the *brb *gene (Figure 2A). Enzymatic activity of the Brb-BgaH fusion protein was detected on agar plates containing X-gal. Colonies of the mutant became blue (Figure 2B). In a control experiment, a mutation eliminating the *brb *start codon (GAG instead of GTG; pVYT2 plasmid) resulted in red colonies (BgaH^-^) on the plates with X-gal (Figure 2C), proving no other potential start codons were active upstream or downstream of the tested *brb *start codon.

Attempts to detect the gene product, Brb, in various WT cell fractions using mass spectroscopy were not successful, possibly due to low levels of this protein, consistent with the low level of *brz*-*brb *transcripts by RT qPCR. The small size and high pI value may also be contributing factors limiting the number of endoproteolytic peptides available for the identification. We also tried to produce samples for analysis that were enriched in Brb. For this purpose, a mutant was constructed which carries an insertion of the DNA fragment encoding the cellulose binding domain (CBD) *in frame *and just upstream of the stop codon of the *brb *gene. No visible band corresponding to a BrbCBD fusion protein was observed on the gels and no BrbCBD fusion protein was found by mass spectrometry, again probably due to low amount of the fusion protein expressed under the native promoter.

### Brb can influence *bop *transcription

**Figure 3:**
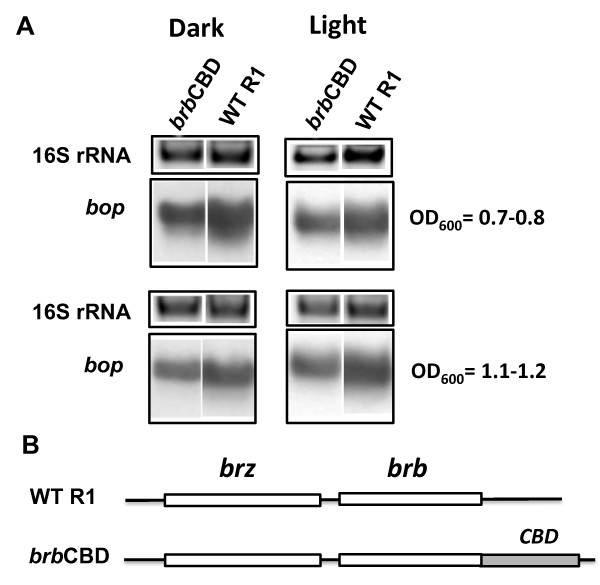
**Effects of CBD domain insertion in the *brb *gene on *bop *transcription**. In panel A, the upper blocks represent the 16S rRNA bands on the agarose gel stained by ethidium bromide. The lower blocks show northern blots of total cell RNA after probing with labeled DNA fragments containing the *bop *gene. Cells were grown in the dark and light. Culture OD_600 _at which RNA was extracted is given at right. WT R1, wild-type strain R1; *brb*CBD, *brb*CBD gene fusion strain. According to densitometry, the levels of *bop *mRNA (normalized to 16S rRNA) from the *brb*CBD mutant corresponded to 67 ± 2% (OD_600 _= 07.-0.8), 70 ± 2% (OD_600 _= 1.1-1.2) of WT in the dark, and 85 ± 3% (OD_600 _= 07.-0.8), 85 ± 2% (OD_600 _= 1.1-1.2) in the light. Panel B shows the locations of mutations in the *brz *and *brb *genes.

**Figure 4:**
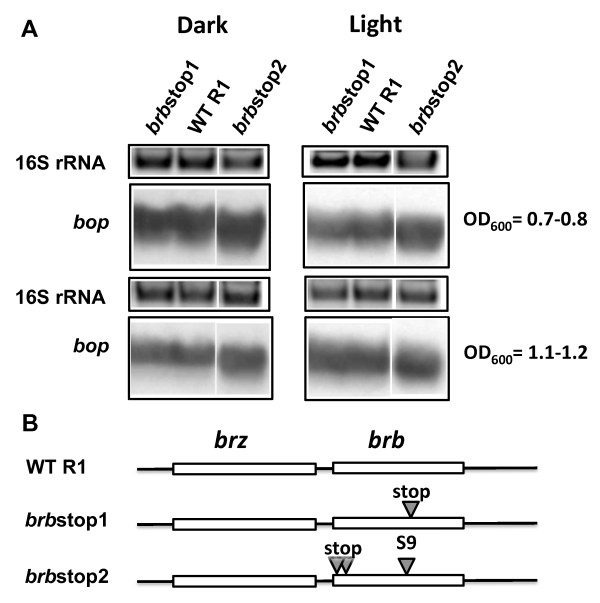
**Effects of inactivation of the *brb *gene translation on *bop *transcription**. In panel A, the upper blocks represent the 16S rRNA bands on the agarose gel stained by ethidium bromide. The lower blocks show northern blots of total cell RNA after probing with labeled DNA fragments containing the *bop *gene. Cells were grown in the dark and light. Culture OD_600 _at which RNA was extracted is given at right. WT R1, wild-type strain R1; *brb*stop1, strain *brb*stop1; *brb*stop2, strain *brb*stop2. According to densitometry, the levels of *bop *mRNA (normalized to 16S rRNA) from strain *brb*stop1 were: 117 ± 5% (OD_600 _= 07.-0.8), 86 ± 4% (OD_600 _= 1.1-1.2) of WT in the dark, and 106 ± 2% (OD_600 _= 07.-0.8), 132 ± 9% (OD_600 _= 1.1-1.2) in the light; from strain *brb*stop2 they were: 175 ± 6% (OD_600 _= 07.-0.8), 95 ± 4% (OD_600 _= 1.1-1.2) of WT in the dark, and 124 ± 9% (OD_600 _= 07.-0.8), 118 ± 11% (OD_600 _= 1.1-1.2) in the light. Panel B shows locations of the mutations in the *brz *and *brb *genes.

Two series of mutants in the *brz-brb *region were produced to demonstrate the involvement of Brb in regulating *bop *transcription. One series used a wild-type *brz *background, and the second used a *brz *inactive mutant (*brz*S9) as the parental background. Although BrbCBD fusion protein was unable to be detected, the *brb*CBD mutant produced less *bop *mRNA and BR than wild-type cells, indicating a role for Brb in *bop *expression (Figure 3; Table 1; Table 2). Given the negative effect of the BrbCBD fusion on *bop *gene expression, the role of Brb was assessed next by translational knockouts. Two knockout mutants having stop codons in the *brb *gene were constructed: *brb*stop1 and *brb*stop2 (Figure 4). The *brb*stop mutants were constructed instead of the *brb *deletion mutant to avoid any possible polar effects of the deletion on transcription of the neighboring genes. The *brb*stop1 mutant had a stop codon in the middle of the *brb *gene and so should produce only the first half of the Brb protein. Mutant *brb*stop2 contained two stop codons at the beginning of the *brb *gene. The *bop *mRNA (Figure 4) and BR levels (Table 1, Table 2), were found to be the same as in wild-type cells in both mutants. The lack of a phenotype could be explained either by the residual activity of a proposed protein complex that regulates *bop *transcription in the absence of Brb protein, or by the ability of the putative complex Bat/Brz to recruit other proteins able to substitute Brb functionally. In the first case, appropriate mutations in the Brb sequence could have more deleterious effects than a knock-out of *brb*. To test this, mutants were constructed based on old observations of changes in *brz *and *brb *that were previously published in a study of *Hbt*. *salinarum *strain S9, long before these ORF's were recognized to be involved in *bop *regulation [3,16]. The S9 strain is a constitutive BR-overproducer, and mutations in Bat were suggested to be responsible for this phenotype [7]. However, if there is a cooperation of Bat with Brz and Brb, mutations in *brz *and *brb *could also contribute to the phenotype in a *bat *wild-type background. Therefore, the naturally selected mutations in *brz *and *brb *of S9 were introduced separately or together into R1 wild-type cells, using the pop-in/pop-out gene replacement method. The *brz *mutations S9 converted Val to Ala, and Ala to Thr located in a region of high homology with Brz from the archaeon *Halorhabdus utahensis *(additional file 2). The *brb *gene mutations S9 resulted in a frame shift and translation of a protein with the N-terminal half of the native Brb followed by a new C-terminal sequence (additional file 2).

**Table 1:** Relative amounts of BR in *Hbt. salinarum *WT R1 cells and *brb*CBD, *brb*stop1, *brb*stop2, *brb*M1, *brz*S9*brb*M2 mutants

	Dark		Light	
**ID**	**OD_600 _= 0.7-0.8**	**OD_600 _= 1.1-1.2**	**OD_600 _= 0.7-0.8**	**OD_600 _= 1.1-1.2**

WT R1	1.20	4.54	1.60	3.94

*brb*CBD	0.20	1.18	0.25	1.00

*brb*stop1	0.66	3.78	1.16	3.46

*brb*stop2	1.24	3.96	1.29	3.91

*brb*M1	1.50	5.01	1.36	4.47

*brz*S9*brb*M2	0.09	0.95	0.00	0.64

For BR measurement cell samples were taken at the same time as for northern blot analysis. BR concentration values are given in μM.

**Table 2:** Comparison of effects of the *brz *and *brb *mutations on *bop *transcription and BR production at OD_600 _= 1.1-1.2

Mutant ID	State of Brz	State of Brb	Effect on *bop*	Effect on BR amount	
			mRNA level	Dark	Light
*brb*CBD	WT	fusion *brb*-*CBD*	down	down	down

*brb*stop1	WT	1 stop codon in *brb*	WT	WT	WT

*brb*stop2	WT	2 stop codons in *brb*	WT	WT	WT

*brz*S9	mutation S9	WT	down	down	down

*brb*S9	WT	mutation S9	WT	WT	WT

*brz*S9*brb*S9	mutation S9	mutation S9	WT	1/2 of WT	1/2 of WT

*brb*M1	WT	mutation M1	WT	WT	WT

*brz*S9*brb*M2	mutation S9	mutation M2	down	down	down

Cell samples were taken at the same time for northern blot analysis and BR measurement.

**Figure 5:**
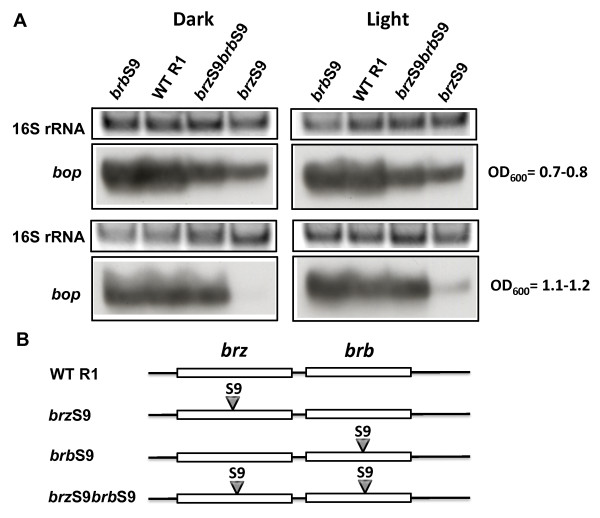
**Effects of S9 mutations in the *brz *and *brb *genes on *bop *transcription**. In panel A, the upper blocks represent the 16S rRNA bands on the agarose gel stained by ethidium bromide. The lower blocks show northern blots of total cell RNA after probing with labeled DNA fragments containing the *bop *gene. Cells were grown in the dark and light. Culture OD_600 _at which RNA was extracted is given at right. WT R1, wild-type strain R1; *brz*S9, strain *brz*S9; *brb*S9, strain *brb*S9; *brz*S9*brb*S9, strain *brz*S9*brb*S9. According to densitometry, the levels of *bop *mRNA (normalized to 16S rRNA) from strain *brzS9 *were: 59 ± 4% (OD_600 _= 07.-0.8), not detectable (OD_600 _= 1.1-1.2) of WT in the dark, and 65 ± 4% (OD_600 _= 07.-0.8), 25 ± 1% (OD_600 _= 1.1-1.2) in the light; from strain *brbS9 *they were: 88 ± 4% (OD_600 _= 07.-0.8), 119 ± 6% (OD_600 _= 1.1-1.2) of WT in the dark, and 135 ± 5% (OD_600 _= 07.-0.8), 147 ± 6% (OD_600 _= 1.1-1.2) in the light; and from strain *brzS9brbS9 *they were: 81 ± 5% (OD_600 _= 07.-0.8), 83 ± 2% (OD_600 _= 1.1-1.2) of WT in the dark, and 86 ± 5% (OD_600 _= 07.-0.8), 115 ± 6% (OD_600 _= 1.1-1.2) in the light. Panel B shows the locations of mutations in the *brz *and *brb *genes.

**Figure 6:**
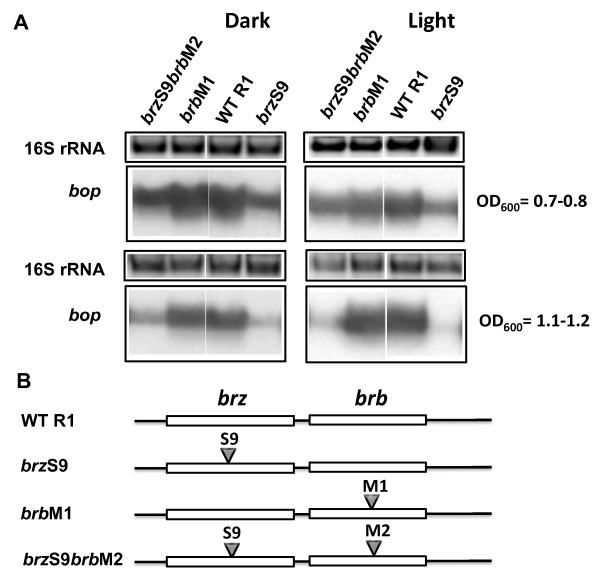
**Effects of M1 and M2 mutations in the *brb *genes on *bop *transcription**. In panel A, the upper blocks represent the 16S rRNA bands on the agarose gel stained by ethidium bromide. The lower blocks show northern blots of total cell RNA after probing with labeled DNA fragments containing the *bop *gene. Cells were grown in the dark and light. Culture OD_600 _at which RNA was extracted is given at right. WT R1, wild-type strain R1; *brz*S9, strain *brz*S9; *brb*M1, strain *brb*M1; *brz*S9*brb*M2, strain *brz*S9*brb*M2. According to densitometry, the levels of *bop *mRNA (normalized to 16S rRNA) from strain *brzS9 *were: 58 ± 1% (OD_600 _= 07.-0.8), 20 ± 2% (OD_600 _= 1.1-1.2) of WT in the dark, and 46 ± 3% (OD_600 _= 07.-0.8), 10 ± 1% (OD_600 _= 1.1-1.2) in the light; from strain *brbM1 *they were: 95 ± 2% (OD_600 _= 07.-0.8), 129 ± 2% (OD_600 _= 1.1-1.2) of WT in the dark, and 82 ± 2% (OD_600 _= 07.-0.8), 105 ± 7% (OD_600 _= 1.1-1.2) in the light; and from the *brzS9brbM2 *they were: 74 ± 2% (OD_600 _= 07.-0.8), 47 ± 2% (OD_600 _= 1.1-1.2) of WT in the dark, and 64 ± 3% (OD_600 _= 07.-0.8), 33 ± 3% (OD_600 _= 1.1-1.2) in the light. Panel B shows the locations of mutations in the *brz *and *brb *genes.

In the initial round, two single mutants, *brz*S9 and *brb*S9 as well as a double mutant *brz*S9*brb*S9 were produced (Figure 5). Northern blot analysis showed the mutated *brb *gene (*brb*S9) had no effect on *bop *transcription if the *brz *gene was from wild-type (Figure 5; Table 2; Table 3). In contrast, cells with the wild-type *brb *gene but mutations in the *brz *gene (*brz*S9) showed down-regulated levels of *bop *mRNA and BR expression (Figure 5; Table 2; Table 3). The effect was more prominent at the stationary (OD_600 _= 1.1-1.2) stage of growth and did not depend on the presence of light. Surprisingly, the double mutant carrying both changes, *brz*S9*brb*S9, showed *bop *mRNA level elevated up to that of wild-type and BR expression up to half of the wild-type level at the stationary stage of growth (Figure 5; Table 2; Table 3). Apparently, the Brb protein can be omitted genetically at the level of the wild-type Brz either completely or with sequences like that of the *brb*S9 mutation. However, at the level of the *brz*S9 mutant knocking out BR regulation, the BrbS9 protein can rescue the S9 mutation of *brz *to restore bop mRNA levels up to that of WT. Since the naturally selected C-terminal sequence of Brb in the *brb*S9 strain shows this compensatory effect, a further two control mutants were produced, *brb*M1 and *brz*S9*brb*M2, with frame shifts changing only the sequence of the C-terminal part of the Brb protein beyond amino acid 23 in an arbitrary manner (additional file 3). The single mutant, *brb*M1, possessed the wild-type Brz, and the double mutant, *brz*S9*brb*M2, carried the S9 mutation in Brz (Figure 6). The phenotypes of both mutants confirmed the dominant role of Brz and specificity of S9 mutations in Brb, as the M2 C-terminal sequence in contrast to the S9 C-terminal sequence in S9 restored the WT phenotype only to a small extent (Figure 6; Table 1; Table 2).

**Table 3:** Relative amounts of BR in *Hbt. salinarum *WT R1 cells and *brz*S9, *brb*S9, *brz*S9*brb*S9, *bp2*stop and *bp4*stop mutants

	Dark		Light	
**ID**	**OD_600 _= 0.7-0.8**	**OD_600 _= 1.1-1.2**	**OD_600 _= 0.7-0.8**	**OD_600 _= 1.1-1.2**

WT	1.20	2.95	1.13	3.16

*brz*S9	0.00	0.18	0.00	0.06

*brb*S9	1.32	3.07	1.43	3.58

*brz*S9*brb*S9	0.27	1.22	0.32	1.59

*bp2*stop	1.75	2.92	1.34	3.32

*bp4*stop	1.13	3.09	1.38	3.25

For BR measurement cell samples were taken at the same time as for northern blot analysis. BR concentration values are given in μM.

Taken together, these results suggest that Brb participates in the regulation of *bop *transcription and, further, that Brz and Brb proteins depend on each other for the activation of *bop *transcription.

### Cooperation of Bat, Brz and Brb in the activation of the *bop *promoter

**Figure 7:**
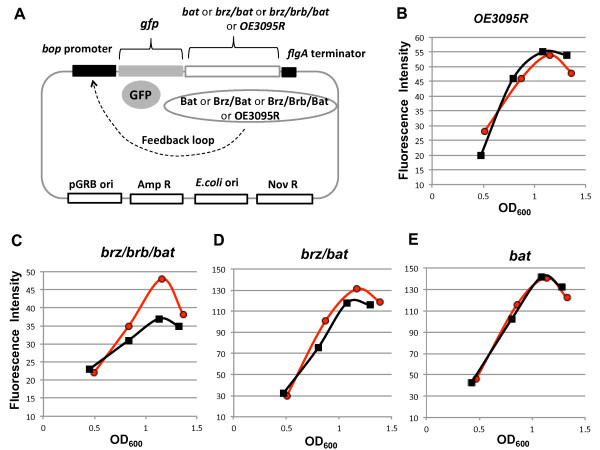
**Activity of the *bop *promoter depending on *brz*, *brb *and *bat *genes expression according to GFP reporter assay**. Panel A represents a map of the GFP reporter plasmid. In panels B, C, D, E kinetic of the *bop *promoter activity during cell growth is shown as GFP fluorescence intensity. The GFP fluorescence is measured in light (red circles) and dark (black squares) for different combinations of the expressed *brz*, *brb *and *bat *genes. *OE3095R*, transformant containing the reporter plasmid with the *OE3095R *gene (B); *brz/brb/bat*, transformant containing the reporter plasmid with the *brz*, *brb*, and *bat *genes (C); *brz/bat*, transformant containing the reporter plasmid with the *brz *and *bat *genes (D); *bat*, transformant containing the reporter plasmid with the *bat *gene (E). In each case at least three biological repetitions were performed. The background level of fluorescence in untransformed cells was constant (around 20 units) during cell growth.

While Bat and Brz are crucial regulators of *bop *transcription, the results described above indicate that Brb is also at least marginally involved in this process. To further test a possible cooperation between the Brz, Brb and Bat proteins, a GFP reporter assay was established. Reporter plasmids contained the *gfp *gene and different sets of the *brz, brb, bat *genes in a single operon under the *bop *promoter control (Figure 7A). At the 3'-end a terminator sequence from the A flagellin operon was added. The reporter plasmids included replication origins of *Hbt*. *salinarum *and *E. coli*, and resistance markers for novobiocin and ampicillin. A control plasmid was identical except for using the OE3095R gene (coding for a conserved hypothetical protein in *Hbt*. *salinarum*, http://www.halolex.mpg.de) instead of *brz*, *brb *or *bat*, since its deletion did not effect *bop *transcription (data not shown). Expression of GFP in these constructs is controlled by the *bop *promoter, allowing the effects of Brz, Brb and Bat on *bop *promoter activity to be directly assessed.

The wild-type cells R1 were transformed by the reporter plasmids and transformants were selected on plates containing novobiocin. Prior to measuring GFP activity, the transformants, grown in medium with novobiocin, were washed and then resuspended in novobiocin-free medium. This was to avoid the negative effects of novobiocin on *bop *promoter activity [19,20]. For each transformant, cells were grown under light or dark conditions, and the fluorescence measured and compared. Plasmid copy number should not depend on the presence and absence of light, so the results for the same transformant should be directly comparable. Since light is an inducer of *bop *transcription, GFP fluorescence would only be expected to be increased in light versus dark conditions by the correct combination of Brz, Brb, Bat proteins expressed from the reporter plasmid. As shown in Figure 7, the enhancement of GFP fluorescence by growth of cells under light was only observed when all three genes: *brz*, *brb *and *bat *were present in the reporter plasmid. This indicates their cooperation for light mediated activation of the *bop *promoter. While the absolute values of GFP fluorescence varied between transformants carrying the same plasmid construct, as well as between transformants carrying plasmids with different constructs of *brz*, *brb *and *bat*, these variations do not effect the interpretation of light versus dark behavior of individual transformants, and were probably due to differences in plasmid copy number. This data clearly identifies a requirement for all three genes, *brz*, *brb *and *bat*, in *bop *induction.

### Homologues and analogues of Brz and Brb in *Hbt. salinarum *and other halophilic Archaea

**Figure 8:**
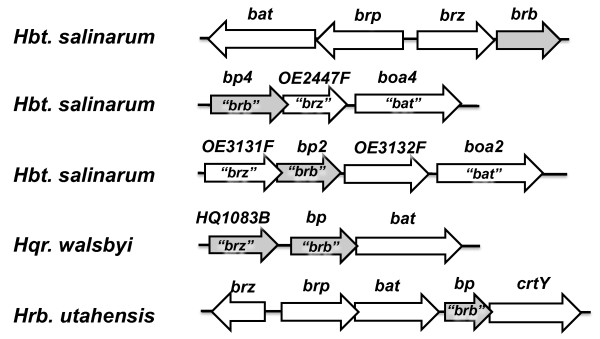
**Organization of *brz*, *brb *and their analogues in *Hbt. salinarum*, *Hqr. walsbyi *and *Hrb. utahensis***. Empty arrows indicate previously annotated genes and grey arrows indicate newly annotated ORFs.

ORFs *OE2447F *and *OE3131F *encode hypothetical proteins that carry zinc finger motifs like Brz, and might also be involved in the *bop *gene regulation. For this reason, searches near these ORFs were conducted to check if they also possessed *brb-*like ORFs encoding proteins potentially able to substitute for the Brb protein. The 5' and 3' UTRs of many genes in *Hbt*. *salinarum *have been determined by Koide; 2009 [18] and this data indicated that transcripts of both *OE2447F *and *OE3131F *were unusually long. The transcription start site (TSS) of *OE2447F *(OE2447F, 62 aa, pI = 4.2) lies 134.51 bp upstream of its start codon, while the transcription termination site (TTS) is located 741.21 bp downstream from the stop codon. The 134.51 bp region, upstream of *OE2447F *contains a small ORF in the same orientation as *OE2447F *(7 bp overlap), and can code for a small, basic protein (45 aa, pI = 9.4) (Figure 8; additional file 4). We designated this protein as basic protein 4 (Bp4). Since the TTS of *OE2447F *is located 741.21 bp downstream from its stop codon, *bp4 *and *OE2447F *are co-transcribed with the *boa4 *gene. Additionally, a TSS for *boa4*, 865 bp upstream of the *boa4 *start codon, also supports co-transcription. For *OE3131F *(OE3131F, 53 aa, pI = 3.9), the analysis of TSS (0 bp from start codon) and TTS (86.8 bp from stop codon) revealed that, in the region 86.8 bp downstream of the ORF, another small ORF is present that overlaps with the 3'-end of *OE3131F *(9 bp) and is predicted to code for a basic protein (33 aa, pI = 12.0). This was designated as basic protein 2 (Bp2) (Figure 8; additional file 5). Thus, besides the Brz/Brb pair, *Hbt*. *salinarum *has the potential to express two similar pairs of small zinc finger and basic proteins, which, together with the Boa4 and Boa2 proteins, could form complexes active in transcriptional regulation.

To test whether Bp2 and Bp4 are functional analogues of Brb, and also influence *bop *transcription, mutants with stop codons aborting translation of the *bp2 *and *bp4 *genes, *bp2*stop and *bp4*stop, were constructed from wild-type cells. The introduced stop codons did not effect BR expression (Table 3). To test a possible substitution of Brb by these proteins, the *brb*stop2 mutant was used as the parental strain to construct a double mutant having stop codons aborting translation of the *bp2 *or *bp4 *genes. Repeated attempts to make such double mutants were not successful even though the same pop-in/pop-out method was used that had readily been able to generate the previous *bp2 *or *bp4 *stop codon mutations. The results indicate that *bp2 *and *bp4 *are not essential by themselves, but may become significant in a *brb *knockout background.

In *Haloquadratum walsbyi*, ORFs encoding a small zinc finger (HQ1083B) and a small basic protein (Bp) were also found upstream of the *bat *gene. The gene encoding the basic protein overlaps with the *bat *gene, so they are likely to be co-transcribed. The *HQ1083B *gene may be transcribed separately or together with *bp *and *bat *genes (Figure 8; additional file 6). The only close homologue of *Hbt*. *salinarum *Brz, is that carried by *Hrb. utahensis*. In this archaeon, the *brz *gene appears to be transcribed as a monocistronic mRNA, but an ORF encoding a small basic protein can be annotated between the *bat *and *crtY *genes (Figure 8; additional file 7). This ORF could either be part of an operon starting from the *brp *and ending just before *crtY*, or transcribed together with the *crtY *gene.

### Novobiocin induces *bop *transcription in *Hbt. salinarum *R1 strain

An inhibitory effect of novobiocin on *bop *transcription was reported for the NRC-1 strain of *Hbt*. *salinarum *[19,20], but its influence in R1 strain is not known. Although experimental care was taken not to allow novobiocin to interfere with transcriptional experiments reported here, we were interested to see the genome wide influences of novobiocin on transcription. Therefore, a DNA microarray analysis of the transcripts from R1 (and R1M1, a bacterioruberin deficient strain derived from the R1) was performed, comparing cells grown in the presence or absence of novobiocin (additional file 8). Surprisingly, *bop *transcription in the R1 (and R1M1) strain was not inhibited, but was 5-fold increased by the presence of 0.1 μg/ml novobiocin. This represents a clear difference to the behavior of the NRC-1 strain, even though R1 and NRC-1 are extremely similar in sequence [21]. It most likely reflects a strain-specific mutation in the B subunit of gyrase in R1 cells (Lys-544-Asn; [21]), as novobiocin binds to and directly affects the gyrase. Attempts to generate an Asn-544-Lys mutant in R1 cells failed, possibly because of the important role of the B subunit of gyrase in recombination events during the pop-in/pop-out procedure.

Besides the novobiocin-induced changes in *bop *transcription, the expression of many other genes were affected, including stimulation of gyrase and type I topoisomerases. These results are similar to the effects described previously in *E. coli*, where topoisomerases are known to be regulated by DNA supercoiling in a negative feedback loop [22]. It has been reported also, that the expression of several heat-shock genes depends on the level of DNA supercoiling [23], and we detected as well an up-regulation of heat-shock gene *hsp20*. Finally, novobiocin exerted an up-regulating effect on genes of the basal transcription apparatus, while some genes encoding ribosomal proteins and many metabolic enzymes were down-regulated (additional file 8).

## Discussion

The current study has shown that besides Bat and Brz there is a third protein, Brb (bacteriorhodopsin-regulating basic protein), involved in regulating *bop *transcription. The *brb *gene is co-transcribed with the *brz *gene, and is predicted to specify a small basic protein. Insertional mutagenesis of *brb *was shown to impair transcription of the *bop *gene and to lower BR expression. In addition, mutations in Brb could complement the effects of Brz mutations, indicating cooperation of the Brz and Brb proteins. At the same time, blocking expression of the *brb *gene by introducing stop codons did not effect *bop *transcription. This would be consistent with an interaction model where Bat and Brz can still form a functional complex without Brb, but a mutated Brb negatively effects function. The latter types of mutants are classified as dominant negative, and commonly reflect alterations in multisubunit complexes. For this type of mutation, null mutations of functionally redundant genes do not alter the phenotype [24]. Redundant genes of Brb are likely to exist in *Hbt*. *salinarum*, as two other small basic proteins (Bp2 and Bp4) are predicted to be encoded by genes found in vicinity of the *boa2 *and *boa4 *genes, which code for Bat homologues. These genes (*bp2 *and *bp4*) were not previously annotated, but they are co-transcribed together with genes encoding small zinc-finger proteins according to published data on the transcription of these genes [18]. Blocking of Bp2 or Bp4 expression did not affect the level of BR expression, but surprisingly, the same pop-in/pop-out approach used for construction of the single mutants failed to produce a double mutant with stop codons inserted in both *brb *and *bp2 *or *brb *and *bp4 *genes. This may indicate such double-mutants have a negative effect on cell growth. Thus, the importance of these proteins as substitutes of Brb in *bop *transcription remains an open question.

Using a GFP reporter assay, we found that light induction of the *bop *promoter takes place only when all three regulating factors are expressed, Bat, Brz and Brb. This indicates that Brb can be a co-activator of *bop *promoter in light (versus dark) when, additionally, oxygen is naturally and gradually depleted during cell growth. Expression of only Brz and Bat in the same conditions did not have a similar activation effect. Thus, the combination of these three proteins can be considered as an optimal set of factors to reach maximum activation of *bop *transcription in light conditions. Similar genes are also found in *Hqr. walsbyi*, indicating this regulatory network is not limited to *Hbt. salinarum *but is distributed among other haloarchaea. It cannot be excluded that this set of proteins (zinc-finger protein, small basic protein, and a flavin-containing protein) might activate transcription not only of *bop*, but also of other genes encoding opsin proteins in haloarchaea. In *Hbt. salinarum*, such genes can potentially be activated not only by Bat, Brz and Brb, but also by a complex comprising zinc-finger protein OE2447F (or OE3131F), basic protein Bp4 (or Bp2) and Bat as a flavin-containing protein. The Bat homologs Boa2 and Boa4 cannot have the same functionality as Bat since, in contrast to Bat, they do not possess a PAS domain containing a flavine binding site.

A marked complexity of *bop *expression at the transcription level has become more apparent from mutational studies of *brz *and *brb*. While only mutations in Bat were previously suggested to be responsible for the Bop-overexpression phenotype of the S9 strain [7], our results demonstrate that mutations in both Brz and Brb can also contribute to this phenotype. Such multiple influences on *bop *transcription are possible if all three proteins form an interacting complex, and where changes in the ternary structure directly affect its ability to influence *bop *transcription. In addition, comparison of *bop *mRNA and BR levels in mutants and wild-type cells points to a tight regulation of both *bop *transcription and translation. BR levels were significantly lower at the early growth phase (OD_600 _= 0.7-0.8), but *bop *mRNA levels were relatively constant, and only slightly higher at a later phase of growth (OD_600 _= 1.1-1.2). This indicates a delay in BR biosynthesis despite the presence of *bop *transcripts. The complex regulatory network controlling BR synthesis probably reflects the huge energy investment required to switch to photosynthetic growth and so provides the cell with the ability to react when conditions are most favorable to do so.

## Conclusions

Based on our results, the activity of *bop *transcription is not only regulated by two major protein factors Brz and Bat, but is also tuned by an additional factor, a small basic protein, Brb. While Brb was not found to be as important as Brz and Bat, a cooperation of Brb with Brz and Bat was demonstrated to provide higher induction of *bop *transcription in response to light.

## Methods

### Strains and growth conditions

*Hbt. salinarum *R1, R1M1 and mutant strains derived from R1 were grown in complex medium as described previously [25], either in the dark or under light. For all experiments, cells were cultivated in 35 ml of medium contained in 100 ml volume flasks, and the flasks closed by aluminium foil. Under these conditions, oxygen in the cultures is naturally depleted during cell growth. The *E. coli *strains DH5α and DH10β were used for transformation according to the supplier's instruction (Invitrogen).

### Construction of mutagenesis vectors and mutants of *Hbt. salinarum*

*brb*CBD, *brb*stop1, *brb*stop2, *brz*S9, *brb*S9, *brz*S9*brb*S9, *brb*M1, *brz*S9*brb*M2, *bp2*stop and *bp4*stop mutants were produced using the pop-in/pop-out method [26]. For this, the plasmids were constructed by inserting of corresponding DNA fragments into the pVT plasmid [10] using *Hind*II, *Bam*HI, *Xba*I, *Pst*I restriction sites. The fragments were produced by PCR using corresponding primers (additional file 9) and verified by sequencing. To create the *brz*S9*brb*M2 mutant, the corresponding plasmid was constructed by cloning the *brz*S9*brb*M2 fragment selected from PCR fragments, amplified by using a primer having a randomly *brb*M2 mutation instead of the *brb*S9 mutation. Transformations were carried out by the PEG method [27,28]. Transformants were selected using blue/red screening [26] by plating the cells onto agar growth medium containing 0.1-0.2 μg ml^-1 ^of novobiocin and 40 μg ml^-1 ^of X-gal [29]. Cells from single blue colonies (pop-in) were propagated in culture medium without novobiocin and plated on agar plates containing 40 μg ml^-1 ^of X-gal without novobiocin. Red colonies (pop-out) were checked for the presence of the respective mutations by sequencing of PCR fragments. For the amplification and sequencing of these fragments the corresponding primers were used (additional file 9). The *brb*M1 mutant was produced as a random mutant during selection of the *brb*S9 mutant.

The *brb-bgaH *mutants were produced in result of the pVYT1 and pVYT2 plasmids insertion in *brz-brb *genes region (Figure 2; additional file 9). The plasmids were constructed by cloning *bgaH *and brb gene fragments into the pAN plasmid [28]. The *bgaH *and *brb *fragments were PCR amplified using corresponding primers (additional file 9). Transformations were carried out by the PEG method [27,28]. Transformants were selected by plating the cells onto agar growth medium containing 0.1-0.2 μg ml^-1 ^of novobiocin and 40 μg ml^-1 ^of X-gal [29].

### Northern blot hybridizations and 5'-3'RACE

Northern blot hybridizations were done as described [28]. DIG-labeled *bop *gene probe generated by PCR were used for the chemiluminescence detection performed with the DIG luminescence detection kit (Roche) according to the supplier's instructions. Densitometry was performed using program ImageJ64. For the *bop *gene PCR amplification primers 1 and 2 were used (additional file 9). Total RNA was prepared from cells, taken in every time series experiment, using the peqGold RNAPure kit (Peqlab Biotechnology) according to the supplier's instruction. For RNA gel electrophoresis 4-5 μg of total RNA were used per lane.

To determine the 5' and 3'ends of transcripts 5'-3'RACE (rapid amplification of cDNA ends) was performed by the use of an RNA circularization mediated method according to Brenneis; 2007 [17]. For cDNA synthesis and PCR amplification from cDNA primers 3 and 4, 5 were used, respectively (additional file 9). The obtained PCR-amplified fragments were cloned (TOPO TA cloning Kit, Invitrogen) and sequenced using M13 forward and M13 revers primers.

### GFP reporter assay

The GFP reporter plasmid, pVTR, was constructed by cloning of following fragments into the pAN plasmid [28]: multiple cloning site, pGRB origin of replication, *bop *promoter, GFP gene (smRSGFP), terminator of A flagellin (*flgA*) operon. The fragments were PCR amplified by using the corresponding primers listed in an additional file 9. The *flgA *terminator has previously been shown to be effective in plasmids described by Furtwängler; 2010 [30]. Different sets of the *brz*, *bat*, *brb, OE3095R *genes were cloned into the pVTR plasmid between the *gfp *gene and the *flgA *terminator. Transformations of wild type R1 cells by the reporter plasmids were carried out by the PEG method [27,28]. Transformants were selected by plating the cells onto agar growth medium containing 0.1-0.2 μg ml^-1 ^of novobiocin. Integrity of the reporter plasmids in the transformants was checked by a restriction analysis of the plasmids isolated from these transformants.

For GFP fluorescence measurement, cells were centrifuged and lysed in 3 ml of buffer (10 mM Tris-HCl, pH = 7.5). For each measurement, equal amounts of cells were used. The absolute number of cells corresponded to 3 ml of culture at OD_600 _= 0.4. The wavelengths of excitation and emission were 588 nm and 610 nm, respectively.

### BR measurement

For BR measurement, cell samples taken in every time series experiment were centrifuged and lysed in 1 ml of buffer (10 mM Tris-HCl, pH = 7.5). For each measurement, equal amounts of cells were used. The absolute number of cells corresponded to 10 ml of culture at OD_600 _= 0.7. The lysates were treated with DNase I for 20 min at 37°C. Absorbance spectra were recorded from 200 to 800 nm and BR concentration was quantified from spectral decomposition performed to estimate the compositions of bacteriorhodopsin-bacterioruberin cell mixtures.

### DNA-Microarray

Total RNA was prepared using peqGold RNAPure solution (Peqlab Biotechnology) from cells grown to an OD_600 _of 0.5. RNA (5 μg) was reverse transcribed into Cy3/Cy5-labeled cDNA using CyScribe First-Strand cDNA Synthesis Kit with enclosed random nonamer primers and Cy3-/Cy5-dUTP (both Amersham Biosciences, Freiburg). Labelled cDNA was hybridized to in-house fabricated whole genome DNA-microarrays [31] at 64°C overnight. To determine the fluorescence ratios the slides were scanned (GenePix 4000 B, Axon Instruments) and the data were extracted using the GenePix Pro 6 software. After background subtraction, pinwise normalization and data evaluation by a Student's T-test, those transcripts displaying a p-value equal or lower than 5 × 10^-5 ^and a ratio of +/- 1.6 were selected as significantly regulated. A detailed description of the microarray design, experimental procedure and data-evaluation is described in [31,32]. The data obtained from the microarray experiment for R1 strain were deposited at http://www.ebi.ac.uk/miamexpress under the accession number (E-MEXP-3219).

## Authors' contributions

VT performed the majority of the experiments (5'-3'RACE, mutant construction and analysis, GFP reporter assay), study design and analysis, drafting the manuscript. RS performed microarray experiments and analysis. KF participated in construction of mutants and their analysis. MDS drafted the manuscript and participated in data analysis. DO conceived study, participated in its design and analysis, drafted the manuscript. All authors read and approved the final manuscript.

## Supplementary Material

Additional file 1 **Genetic map of the genes and open reading frames in the *bop *gene region**. Numbers indicate chromosomal coordinates of the genes and ORFs. Click here for file

Additional file 2 **Comparison of Brz, *brz *and Brb, *brb *sequences from strains of *Hbt. salinarum *R1 **[21], **S9 **[16]**and *Hrb. utahensis***. Red letters correspond to mutations. Click here for file

Additional file 3 **Comparison of Brb and *brb *sequences from *Hbt. salinarum *wild-type strain R1 and S9, *brb*M1, *brz*S9*brb*M2 mutants**. Red letters correspond to mutations. Click here for file

Additional file 4 **Operon organization of the *bp4 *and *OE2447F *genes of *Hbt. salinarum *R1**. The protein sequences are given below to nucleotide sequences. The arrow above the nucleotide line indicates the neighboring genes. Underlined letters correspond to translation start codons and boxed letters are translation stop codons. Click here for file

Additional file 5 **Operon organization of the *OE3131F *and *bp2 *genes of *Hbt. salinarum *R1**. The protein sequences are given below to nucleotide sequences. The arrow above the nucleotide line indicates the neighboring genes. Underlined letters correspond to translation start codons and boxed letters are translation stop codons. Click here for file

Additional file 6 **Organization of the *HQ1083B *and *bp *genes of *Hqr. Walsbyi***. The protein sequences are given below to nucleotide sequences. The arrow above the nucleotide line indicates the neighboring genes. Underlined letters correspond to translation start codons and boxed letters are translation stop codons. Click here for file

Additional file 7 **Organization of the *bop *related genes cluster of *Hrb. utahensis***. The protein sequences of the *bp *gene are given below to nucleotide sequences. The arrow above the nucleotide line indicates the neighboring genes. Underlined letters correspond to translation start codons and boxed letters are translation stop codons. Click here for file

Additional file 8 **DNA microarray analysis of differentially expressed genes from *Hbt. salinarum *R1 and R1M1 cells grown in the presence of novobiocin**. Click here for file

Additional file 9 **Primers used in this work**. Click here for file
