# Supplementary figures and images for: A small basic protein from the brz-brb operon is involved in regulation of bop transcription in Halobacterium salinarum
Source: BMC Mol Biol. 2011 Sep 19;12:42. doi: 10.1186/1471-2199-12-42 (PMC3184054; doi:10.1186/1471-2199-12-42)

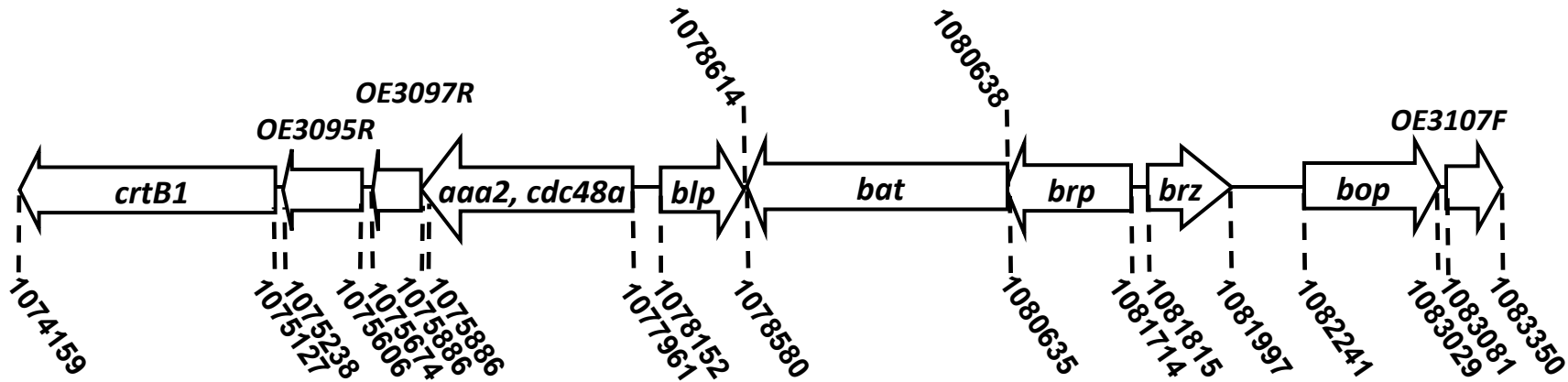

Supplement: Additional file 1 — Genetic map of the genes and open reading frames in the bop gene region. Numbers indicate chromosomal coordinates of the genes and ORFs. [file 1471-2199-12-42-S1.PDF]
