# Supplementary material for: A small basic protein from the brz-brb operon is involved in regulation of bop transcription in Halobacterium salinarum
Source: BMC Mol Biol. 2011 Sep 19;12:42. doi: 10.1186/1471-2199-12-42 (PMC3184054; doi:10.1186/1471-2199-12-42)
Supplement: Additional file 2 — Comparison of Brz, brz and Brb, brb sequences from strains of Hbt. salinarum R1 [21], S9 [16]and Hrb. utahensis. Red letters correspond to mutations. [file 1471-2199-12-42-S2.PDF]

Brz

```
Hbt.salinarum R1  1 MPITDLHCPRCGSDVKMGLPMGATVKSVTAASRQEPTSDTQKVRTVECRNDHEFFVRFEW  60
Hbt.salinarum S9  1 MPITDLHCPRCGSDVKMGLPMGATVKSATTASRQEPTSDTQKVRTVECRNDHEFFVRFEW  60
                    M I  L CP CG+  +MGLP   TV+SVT   R+EP  D  KVR   C N HE +V F +
Hrb.utahensis     1 MGIEQLSCPACGATFEMGLPRDVTVQSVTTEDREEPDDDRVKVRPNACSNGHECYVMFRF  60
```

brz

```
Hbt.salinarum R1  1 GTG.....................GTCACGGCT.....................TAA 183
Hbt.salinarum S9  1 GTG.....................GCCACGACC.....................TAA 183
```

Brb

```
Hbt.salinarum R1 1 MHASTSPRVFRRQPAVEPAGSGHFTAAVAHTLGGVRYFGMVWNPRVGSVSDGSSV  55
Hbt.salinarum S9 1 MHASTSPRVFRRQPAVEPAGSGHLQPLWPKWWGALFWYGLESACRLRV         48
```

brb

```
Hbt.salinarum R1 1 GTG...........TCACAGCCGCTGTGGCCCACACACTCG..........TAA 168
Hbt.salinarum S9 1 GTG...........T-ACAGCCGCTGTGGCCCA-A-A-T-G......TGA     147
```
