# Supplementary material for: A small basic protein from the brz-brb operon is involved in regulation of bop transcription in Halobacterium salinarum
Source: BMC Mol Biol. 2011 Sep 19;12:42. doi: 10.1186/1471-2199-12-42 (PMC3184054; doi:10.1186/1471-2199-12-42)
Supplement: Additional file 3 — Comparison of Brb and brb sequences from Hbt. salinarum wild-type strain R1 and S9, brbM1, brzS9brbM2 mutants. Red letters correspond to mutations. [file 1471-2199-12-42-S3.PDF]

```
Brb R1 1 MHASTSPRVFRRQPAVEPAGSGHFTAAVAHTLGGVRYFGMVWNPRVGSVSDGSSV         55
Brb S9 1 MHASTSPRVFRRQPAVEPAGSGHLQPLWPKWWGALFWYGLESACRLRV                48
Brb M1 1 MHASTSPRVFRRQPAVEPAGSGHFTAAVAQMVGCAILVWFGIRVSAPCLTVHRSKFRHERTILIGS 66
Brb M2 1 MHASTSPRVFRRQPAVEPAGSGHLQRCGPNGGVRYFGMVWNPRVGSVSDGSSV          53
```

```
brb R1 1 GTG..................TCACAGCCGCTGTGGCCCACACACTCG..........TAA        168
brb S9 1 GTG..................T-ACAGCCGCTGTGGCCCA-A-A-T-G....TGA              147
brb M1 1 GTG..................TCACAGCCGCTGTGGCCCA-A-A-T-G...............TAG   201
brb M2 1 GTG..................T-ACAGC-GCTGTGGCCCA-A-A-T-G.........TAA         162
```
