# Supplementary material for: A small basic protein from the brz-brb operon is involved in regulation of bop transcription in Halobacterium salinarum
Source: BMC Mol Biol. 2011 Sep 19;12:42. doi: 10.1186/1471-2199-12-42 (PMC3184054; doi:10.1186/1471-2199-12-42)
Supplement: Additional file 4 — Operon organization of the bp4 and OE2447F genes of Hbt. salinarum R1. The protein sequences are given below to nucleotide sequences. The arrow above the nucleotide line indicates the neighboring genes. Underlined letters correspond to translation start codons and boxed letters are translation stop codons. [file 1471-2199-12-42-S4.PDF]

TCA--*phiH1*-CATGCAAATCTGTACGCAGTGCCTCTCGGATTACTATTTTCATCAC

GATGAAGGCGTGTACAATAACCAATGTTATGTGTACAAGACTGTCGGTAAGCACTAA

bp4

GTGGCTTGCCTACAGCATTGTCGGCTACAGGAGCGATTCGAGAGGCGACCAACCTAT
M A C L Q H C R L Q E R F E R R P T Y

CGCCTCGAAAAGTGCGAGCCTCGCTGCTGCAACAGCAGGCTCGCGCGGGCTCCTGTA
R L E K C E P R C C N S R L A R A P V

CCGCAGCTACAGAAGCCATGCTAGAGTCCACCGACGACGCTGAAGAGCGTTCCGACG
P Q L Q K P C

OE2447F

M L E S T D D A E E R S D
GCGCACAGCATTTCGACTGTCCGAACTGCGGCGGGGGTGCAGGCGGTCACACAGC
G A Q H F D C P N C G R G V Q A V T Q

ACGGGCCGACCGACGCCACCGCCAGCCCGTGCGGGTGTTCGATCAGCCCCGCGCACA
H G P T D A T A S P C G C S I S P A H

TCGACGCCCTCATCGCCGGGCGAGACGACGACTGACGCCGCCACGCCGCGACGCCGC
I D A L I A G R D D D

CCGCCAGCGGGCTGCGTCGCCGGGCTCGCGCCCCCGTCAGCCCGGCGCAGTCGGCGC

GGACGACCACGCAAGGTTGATGGTTCGGGATTCGAAATAATCACGAAGT

ATG--*boa4*--TAA
