# Supplementary material for: A small basic protein from the brz-brb operon is involved in regulation of bop transcription in Halobacterium salinarum
Source: BMC Mol Biol. 2011 Sep 19;12:42. doi: 10.1186/1471-2199-12-42 (PMC3184054; doi:10.1186/1471-2199-12-42)
Supplement: Additional file 6 — Organization of the HQ1083B and bp genes of Hqr. Walsbyi. The protein sequences are given below to nucleotide sequences. The arrow above the nucleotide line indicates the neighboring genes. Underlined letters correspond to translation start codons and boxed letters are translation stop codons. [file 1471-2199-12-42-S6.PDF]

```
TGA--capC—CATAATGATACTGACAACAATCAATAGTAGATACTTTCTCCATCAT

TCGTATTTATTTGCAAGATAATAAATTCATTCTTAATCTCATATTAATATCAAAACA

TAAAATGTAAAATTAATGCTAGCAGCAGTTATTTTATTCCGTCAGTTGGTATTGATA

CAATACACTCAGTATTCAACCTGATTTCATCTCATTCATCAGGGAGCGAATCCTTCA

HQ1083B
TAGATGTGCAGAGTGCCCGTTTCTTCTGGGTTCACATACACGTACTCGTCCATTCTG
    M  C  R  V  P  V  S  S  G  F  T  Y  T  Y  S  S  I  L
CAAAGAAGCTCAATTTCTTTCCTCAATCAAAATCACGGCATCCATGAAATGAGTTCG
 Q  R  S  S  I  S  F  L  N  Q  N  H  Q  I  H  E  M  S  S
ATATCTTACTCACATCGCTTATGTGTCATGTTCATTCACACTACATGCGTCTCACAT
 I  S  Y  S  H  R  L  C  V  M  F  I  H  T  T  C  V  S  H
ATCGTGTATTAACTATAAATCATTTAACTTTCGTCAGCAAAACCAACTACTTTCGCA
 I  V  Y

bp
AACGTTAGCATTATACCTCAGGTGCTCGTGATGCAATTAGTGACAAAATCAGTGGAT
                        M  L  V  M  Q  L  V  T  K  S  V  D
TCTCAGTTATACCCATCACGGTGGGTATCGCTGCAGCGAGTAATCTTATTAATAAAA
 S  Q  L  Y  P  S  R  W  V  S  L  Q  R  V  I  L  L  I  K
AAATCAATTATCGACGTTGCAATCGTCATGAGAATTGCGTACAATCTAACTGAATCC
 K  S  I  I  D  V  A  I  V  M  R  I  A  Y  N  L  T  E  S
AGAGGTCAGAGATAATG--bat--TAG
 R  G  Q  R
```
