# Supplementary material for: A small basic protein from the brz-brb operon is involved in regulation of bop transcription in Halobacterium salinarum
Source: BMC Mol Biol. 2011 Sep 19;12:42. doi: 10.1186/1471-2199-12-42 (PMC3184054; doi:10.1186/1471-2199-12-42)
Supplement: Additional file 7 — Organization of the bop related genes cluster of Hrb. utahensis. The protein sequences of the bp gene are given below to nucleotide sequences. The arrow above the nucleotide line indicates the neighboring genes. Underlined letters correspond to translation start codons and boxed letters are translation stop codons. [file 1471-2199-12-42-S7.PDF]

TCGGTATAGTCCACGGGTTGGACCAGTGAAACCCTGTCGGTACGTGCGACCTTCGCT

GAATTGCCACTGGTTGTACCAAATGAATTGGGTACGACCATTATATCAATCCCAGGA

CAATTCCTTATGTGGCATG--*bop*--TGAGCTGTACAGTGCCGGAAGTCCAAGTCGA

ATTTCGAACTTTTTTCAAGGTTGCAGTCGCCGAGAAGTCACGCAACTTCGTCCGCGA

GCGATCACCTA--*ctrY*--TGTCATCGCGAGTGCCCCATCCGATGACCGACGACACC
R S H G M R H G V V G

bp

GGTACTTGTCTTCCTGGGGGCAGTAGCCGGTCCTGGAAGGTTCGGGGGCTCGATTCT
T S T K R P A T A P G P L N P P E I R

CATGAGCAGTCTCTCATTGTACGTATGCCAGCCGGCTGAAGTCGATACCTATTCTGT
M

TGGGGTACCGACCGGATCGAACGGCTACCGTGCCATCGATTACCGG

TCA--*bat*--CATGGCCACTGTGTTGGCAGACGATTACCGTAAAGCTGGGGCTCG

AATCGACTGATACCCGGCTGGTCGGCGGAGCTCA--*crtI*--TCAT--*brp*--CAT

GGGCCGGGGTTCGCCCGGCAGAGTCCTCAAGTACCCGCCCAACTCGTTTGGGTCCC

CTTCCGGGACCAGCCACGATCGATGGCCGGCGATGACAGACCATTTATTTCGCCGG

GGGACTACCTGGGGGATAATG---*brz*---TGA
